# Veiled agency? Children, innovation and the archaeological record

**DOI:** 10.1017/ehs.2021.9

**Published:** 2021-02-09

**Authors:** Kim Sterelny

**Affiliations:** School of Philosophy, ANU, Australia

**Keywords:** Forager childhood, forager social learning, forager innovation, children and innovation, archaeology of children

## Abstract

Children and subadults were obviously part of ancient human communities, and almost certainly, in important ways their activities were distinctive; they did not routinely act like scaled down adults. Yet their presence was quite cryptic, but not entirely hidden. Their lives and acts did leave traces, although these tend to be be fragile, ambiguous and fast-fading. In addition to pursuing the methodological issues posed by the detection of subadult lives, this special issue raises important questions about the role of children, and their willingness to experiment and play, on innovation. It is true that ethnographically known forager children are almost certainly more autonomous, experimental and adventurous than WEIRD children, and this was probably true of the young foragers of the early Holocene and late Pleistocene too. Their greater willingness to experiment probably fuelled a supply of variation, and perhaps occasionally adaptation as well, especially finding new uses for existing materials. Much more certainly, innovations tend to be noted, taken up and spread by adolescents. They were vectors of change, even if perhaps only rarely initiators of change.

## Graphical abstract

**Figure:**
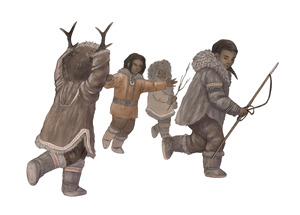

**Social Media Summary:** Forager children were freer than the regimented children of the West, and so were important in spreading, and perhaps initiating, change.

## 1. Two themes: methodology and innovation

This special issue revolves around two themes. The first is methodological. Children (and perhaps more generally subadults) were obviously part of ancient hominin populations, and active participants in their social and economic lives as well. Those activities were likely to be distinctive: subadults were not just physically miniaturised versions of adults in body or behavioural profile. Differences in size, strength, informational capital, social capital and motivation imply quite fundamental differences in daily routines. The methodological theme addresses the extent to which these subadult activity patterns are archaeologically visible, or can be made archaeological visible. For the most part, in this special issue there is a strong reliance on uniformitarian principles, relying on contemporary data to construct and confirm robust patterns in the ways children and adolescents live their lives, and using those to interpret otherwise rather enigmatic historical traces. In turn, this tends to impose fairly shallow time depths, restricted to the Holocene and terminal Pleistocene. The material traces of ancient children's lives tend to be subtle and fragile. Their bones are lighter and less durable; their distinctive activities do not leave long-enduring traces like hearths, middens and the like. Moreover, contemporary children are increasingly imperfect guides to behaviour and life history parameters in the past, as we consider more and more ancient juvenile hominins.

The second theme concerns innovation. Over time and space, there is very considerable variation in the complexity and diversity of hominin material culture, and hence in the pace of innovation (Kuhn, 2020). This variation over space and time has variously been ascribed to changes in individual cognitive capacity (Klein, 2008 – although in this case with a focus on change over time); to change and variation in the environment (for example, complexity and diversity is often taken to be a response to risk; Torrence, 2001); and to change in the demographic character of evolving hominin populations (Powell et al., 2009). Very likely, all three factors have played some role. This issue explores a potential fourth factor: the length and character of subadult life. A standard view of anatomically modern human (AMH) life history holds that our long subadult span is an adaptation to allow children to absorb more of adult-created and adult-curated cognitive capital. This special issue probes the hypothesis that children and adolescents contribute to cognitive capital; they do not just inherit it.

## 2. Identifying children, their roles and their activities

Fossilisation is a rare process, but the fossil record of hominins is surprisingly rich, perhaps as a result of the wide distribution of our lineage. Children are less likely to fossilise than adults; their bones are lighter and more fragile. Even so, children and adolescents are part of that record, although not a rich part. Sian Halcrow and colleagues estimate the existence of somewhere between 200 and 300 hominin juvenile fossils covering the 7 million years or so of hominin history (Halcrow et al., 2020). Nowell and French, estimating the traces of adolescents in European Upper Palaeolithic, give a range of 50–80 individuals, with many of these just fragments, and often with age range estimates that extend forward into young adulthood and backward into childhood. So the physical record is sparse. Even so, Halcrow and colleagues suggest that this record is informative about patterns of care, especially in tough times, in the population they represent. The intense nutritional demands on mothers and infants in fuelling growth will expose otherwise cryptic subsistence and social stresses in these populations. Such stresses leave detectable traces on bones and teeth. Conversely, if infants and children show few traces of dietary or other deficiencies, that suggests that, even in the deep past, forager communities managed subsistence risks reliably.

Rather more ambitiously, they suggest that the archaeological record includes indirect signals of the cognitive and emotional traits required to sustain infant care. The idea is that care for the seriously ill or disabled requires the same skill set as infant care, and so the archaeological signature of such care is evidence of the active presence of this skill set in the community. Such cases exist:

Presenting a case study of an individual with paleopathological evidence for quadriplegia in prehistoric Vietnam, Tilley and Oxenham (2011) argue that the survival and good mental health of a person with a serious disability necessitated the provision of long-term, skilled and consistent care, likely involving multiple group members, including the allocation of food/a special diet, water, shelter, bedding, a hazard-free environment, help with eating and drinking, and managing hygiene (removal of wastes, bathing). Interestingly, if we consider human infant care, infants have the same requirements as an individual with a significant disability, and in addition require breastfeeding, special preparation of foods (such as the pre-mastication of food), holding, carrying, rocking, sleeping, massaging, and assistance to keep cool/warm. (Halcrow 2020)

We will return to indirect inference in discussing baby slings. However, this indirect inference is both redundant and too ambitious. The case study is from the Neolithic Vietnam, about 4000 years ago, and it is the oldest known clear case (although there are older suggestive ones). Infant care must be much more ancient; human infants were at least somewhat altricial before the evolution of AMHs, and AMH babies and infants are appallingly useless. We do not need a Neolithic example of disability care to show that ancient humans were capable of recognising and meeting the needs of their helpless young. Moreover, the case study is of an individual needing care over a much longer period: 10+ years. One lesson of forager ethnography is that forager children become self-reliant, en masse, remarkably early (Lew-Levy et al., 2020b). Furthermore, parents can reasonably hope to get a return on their investment: they can reasonably hope for material and emotional support from their children as they, the parents, come to need it. One striking feature of this Neolithic example is that at some point the carers almost certainly realised that he was never going to be well enough to reciprocate. Finally, and perhaps most important of all, infants are not helpless because they are diseased. Their helplessness is accepted as a normal though transient condition. This is in contrast to illness. Many cultures assume that serious illness is the result of malign witchcraft (see for example (Knauft, 2005). No culture thinks that infants are helpless as the result of malign witchcraft. So the ideology of disability care is very different from that of infant care.

Indirect inference is central to Suddendorf et al. (2020), for they aim to identify the emergence of a cryptic technology, containers. A particularly salient container is the baby sling (and its variants), as this eases the burden of carrying babies and infants. If mothers had to forage on a regular basis while actually holding a baby or infant, their resources would be brutally taxed. This is a cost of combining bipedality with altricial infants. That cost is high, so one suspects that something sling-like was invented early. Yet the direct evidence of containers like baskets and nets is very recent; about 30,000 BP (Suddendorf et al., 2020). However, until the invention of pottery, almost all containers were made from soft materials. Even if Mid Pleistocene baby slings were in routine use, we would be most unlikely to find their traces. Yet as Suddendorf et al. also note, the fact that baby slings would be enormously useful is no evidence of their use.

Can we move beyond a plausible guess? One possible route forward is to identify ‘container signatures’: behaviours with material traces that would be impossible or prohibitively uneconomical without containers. One uncontroversial container signature is a substantial sea crossing. Boats themselves count as containers, and boat travel would be unviable without containers to store water (and probably food and various small items of equipment). However, sea crossings are also relatively recent, even with the dates of Australian colonisation now pushed back to about 65 kyr. Arguably, central place foraging is itself a container signature, for it is likely that the strategy of gathering and returning to home base would be energetically unsustainable without some form of container technology. If foragers gathered low-value resources and returned with them held in their hands, quite likely the rewards would not pay the costs of foraging. This seems particularly pertinent to the standard version of the grandmother hypothesis (O'Connell et al., 1999): if grandmothers foraged at a distance and returned to home base with Underground Storage Organs (USOs), those USOs were not hand-held. So if there were strong independent support for this version of the grandmother hypothesis, that would provide indirect evidence for erectine female container use. Such an indirect inference would gain further support from the fact that there are natural incremental pathways to container manufacture: first noticing and exploiting natural containers (gourds full of rainwater, natural depressions in rock, perhaps animal skins or even the intestines of large animals), then keeping and curating such containers, then searching them out, then making them. Such natural pathways strengthen the claim that container technologies are not just useful, but also within the easy cognitive reach of middle Pleistocene hominins.

However, even if erectines had containers, they may not have had slings. While we might guess that slings are an especially simple container, our intuitive judgements of simplicity are not reliable. Yet a simplicity claim could be supported experimentally. Suddendorf et al. note that great apes, mostly in captivity, from time to time use containers spontaneously. If experiments were to show that great apes, with fairly minimal environmental structuring, can learn to make and use a sling, that would support the idea that sling technology was within the cognitive reach of fairly early hominins like habilines. Habilines were somewhat more encephalised than great apes, and they showed in their lithics at least somewhat greater facility in tool manufacture and use.

So far, the discussion has focussed on signals of adult care of children, not children's own role as active agents. A cautionary tale emerges from a recognition of that active role. Children's material culture and niche constructing can easily be misread as evidence of ritual behaviour. Figurines, including human–animal hybrids, may be dolls or toys rather than totems. That is true even if they were well made. Especially in transegalitarian societies, children's material culture may well include carefully crafted items made from expensive materials (see in particular the discussion and examples in Lbova, 2021). Likewise, secret places, with evidence of fire and other human activities, but without domestic detritus, may be children's spaces. As Michelle Langley and her collaborators show, cross-culturally children like to find and modify their own spaces, away from adult eyes. They also like to collect and curate unusual objects (Langley, 2020; Langley & Litster, 2018). Langley identifies plausible examples of children's spaces (Langley, 2020). Her examples are close to adult domestic sites, as are most of the ‘play tipis’ identified in Mackenzie (2020). However, distance from adult domestic sites may not be a reliable way of excluding the agency of children: ethnography shows that forager children have far more freedom to explore away from adult eyes, despite possible dangers, than children in Western Industrial Rich Democratic (WEIRD) societies (Boyette & Hewlett, 2018; Lew-Levy et al. 2017). Expertise is a more reliable signal: the deep cave art sites of glacial Europe are not children's spaces. Identifying children's spaces relies on ethnography and developmental psychology (and hence only from data about contemporary AMHs), moreover these sites are fragile, as small groups of transient children rarely leave enduring traces. So the temporal scope of these analyses is inevitably restricted: it is very difficult to show the existence of children's spaces in the deep past. The convincing examples of their special places and their material culture are relatively recent. Even so, cautionary implications have a much deeper relevance, for instance warning against over-interpreting supposedly ancient figurines like the Berekhat Ram. However, this special issue has ambitions that go well beyond cautionary tales in linking children to innovation. That is the topic of the next section.

## 3. Children as innovators?

In considering the papers that connect children's innovation to variation in innovation and technology in the archaeological record, it is important to distinguish four hypotheses about children and innovation:

H1. Children generate information and/or technique that is new to them. They acquire know-how by discovering it for themselves, rather than acquiring it from their social group.

H2. Children, and more specifically adolescents, are a primary vector through which an innovation made in one generation, and in one particular residential group, establishes in the next generation and over a broader social network.

H3. Through their social learning strategies, children generate variation around current practices in their social group, and some of that variation may become fuel for further adaptive change.

H4. Children generate information and/or technique that is new to their community.

Experimental and descriptive materials mostly support H1, although there is some direct ethnographic support for H2. However, it is H2–H4 that are relevant to big picture explanations of variation in the complexity and diversity of material culture over space and time. A pivotal issue, then, is the extent to which the truth of H1 is evidence in favour of H2–H4.

The case for H1 itself is persuasive: the ability of children to explore and discover for themselves has been under-estimated, in part as a result of an experimental tradition in developmental psychology. These experiments seem to show that children are remarkably poor at innovating. Children under 10 rarely pass a test that requires the manufacture of a hook from pipe-cleaners to fish an object out of a tube. Likewise, young children rarely succeed in assembling a stick to push an object out of a tube. Children under 10 were a little more successful, but not much more successful, at a social task, one that required them to invent a new sign on the fly (Lister et al., 2020). These results may well be misleading. Lew-Levy and colleagues point out that they probably understate the innovation potential even of WEIRD children, given that the test is time-pressured. Moreover, many children under 10 will have had little experience exploring the affordances of pipe cleaners. Few children under 10 smoke a pipe (although in some WEIRD countries, pipe-cleaners do double as a play material). Additionally, these children were tested in isolation, thus ruling out collaborative learning and innovation. More importantly, forager children are likely to innovate much more than WEIRD children, given that they grow up in an environment in which they have a lot of autonomy to play, to experiment and to explore the affordances of the material substrates to which they have access. In many forager cultures, experimental learning for oneself is positively encouraged. Furthermore, they routinely engage in peer-peer learning. While adults support social learning, they do relatively little directive teaching. Instead, they scaffold learning with equipment and raw materials, they provide occasional advice and they allow children to involve themselves with adult activities. Finally, children, even quite young children, do not just imitate in play adult economic and social activities, they practice those activities. They engage in subsistence activities, often in distinctive ways. So if they were tested in ecologically valid contexts, Lew-Levy and her colleagues conclude that forager children would innovate much more than WEIRD children, even allowing for the fact that WEIRD children's capacities are probably understated (Lew-Levy et al., 2020b).

The case is persuasive, and if it is correct, there is good reason to expect that this conclusion would extend to earlier Holocene and late Pleistocene forager children too. For one thing, it is a remarkably efficient education system, reliably producing efficient adult foragers while imposing low opportunity costs on adults. Moreover, as Lew-Levy et al. (2020b) show, there is archaeological evidence of the material and social scaffolds familiar from forager ethnography. These include miniature weapons and tools (and perhaps even the over-size bifaces occasionally found). Some were probably usable versions of their adult equivalents, others were more clearly toys. In addition, there are lithic work sites plausibly interpreted as teaching sites (although always with some uncertainty). These include cases highly expert knappers seem to be working along side others with much lower skill levels. In addition, there is evidence from cave sites of children being part of communal activities, not excluded from adult worlds. We have very limited archaeological insight into the learning environment of the Holocene and late Pleistocene. However, to the extent that we have traces of that environment, those traces suggest an education system broadly similar to that known from forager ethnography.

However, while H1 is well supported, prima facie that offers little support for H4. In general, forager residential groups have worked their territories for generations, and the simple, low-skill variants of their standard operating procedures will mostly have been found quite quickly. The preceding generations were just as experimental, just as free to play and explore, as the focal generation of children. There are many ways to skin a cat, but the options are not limitless, and the easy alternatives to the typical local cat-skinning practices will soon be explored. Except perhaps in highly dynamic environments generating new opportunities, or in newly colonised ones, there is little reason to expect childhood play and experimentation to find adaptive tweaks to existing practices.

Two papers in this issue resist this sceptical take on H4. First, Riede et al. (2021) suggest that the extent to which communities invest in children's play correlates with the dynamism and richness of their material culture. They develop this suggestion through two intriguing case studies. One is the contrast between Palaeo-Eskimo and Neo-Eskimo (especially the Thule) material cultures on Greenland. The other is the invention and proliferation of the wheel in West Eurasia. Greenland was occupied in two distinct temporal waves separated by about 500 years: an initial sequence of Palaeo-eskimo cultures that lasted a couple of thousand years (contracting to a remnant but hanging on until about 700 AD). A second wave (the Thule culture) began about 1200. Making all due allowance for preservation biases, the palaeo cultures show few signals of innovation in their material culture, and nor do they show the archaeological signatures of children's material culture, of toys and other miniatures. In contrast, the Thule are highly innovative, with the umiak (a larger, more stable version of the kayak), more diverse harpoon forms and more kayak and clothing designs. Moreover, their children have a rich material culture, with evidence of both space of their own and of toys and other miniatures. This is typical of ethnographically known eskimo cultures. That ethnography further suggests that these cultures scaffold their children's social learning more through material supports than by explicit teaching.

Along similar lines, Riede and his colleagues suggest a similar connection between children's material culture and technical innovation, with the break-through technology of the wheel. That technology seems to arrive fairly late. The early dates for a full-fledged wheel are around 3500 BCE. However, there is a very intriguing possibility that the first archaeological signal of wheeled objects were toys (or ritual objects) from Tripolye, in what is now the Ukraine. This site was both economically and technically dynamic, and one which would have rewarded innovation in transport technology. Between 4100 and 3600 BCE, it became a mega-site with a population in the tens of thousands. It was several hundred hectares in size, with large-scale pottery production and ox-drawn sledges. So there was specialisation, trade and transport of high volume, heavy goods, but over a short range. Road making would have been worth the trouble. So at Tripolye, there were presumptive miniature toys with axles and wheels. There were domesticated oxen trained to pull a wagon-chassis on runners. Inventing a true wagon required up-scaling a toy technology and combining these ingredients. Of course, two examples prove nothing, but let us suppose there is a causal connection between the material scaffolding of children's development and the dynamism of adult material culture. That leaves four possibilities open:

P1: Common cause. The Thule (and similar cultures) value and take pride in artisan skills and their products. This leads (a) to their heavily investing in their children's craft education through the provision of material scaffolds, and (b) to high levels of innovation.

P2: Toys are the leading edge of technology. In cultures that value artisan skills and their products, toys are an inexpensive form of experimentation. Toy-making keeps both time and raw material costs low. In general they can be made at leisure, not to any strict timetable. So their opportunity costs are low too. Every now and again, experimental toy-making will lead to innovations in technique or product, as in the wheel.

P3: The benefits of education. Material scaffolding of the kind practised by the Thule (and perhaps the Tripolye) is an extremely effective way of supporting learning. Children are highly motivated to learn, and their learning is supported in ways that make learning successful. As Thule children come out of adolescence, many of them are confident and skilled, and this expertise (in rewarding environmental contexts) leads to higher rates of successful innovation.

P4 (a special case of H4): Subadult innovation. Children whose skill acquisition is supported through material scaffolding have the freedom to experiment and the skills and raw materials that make such experiments occasionally successful, not just for them, but for their community.

In considering the wheel, Riede et al. (2021) opt for P4:

the co-existence of an animal-drawn (non-wheeled) form of vehicle and wheeled miniature items presented preconditions and a latent potential for developing full scale wheeled vehicles. However, the two needed to be combined creatively, and in this process inquisitive and entrepreneurial children and adolescents could plausibly have played the key role. Youngsters in this cultural niche had likely observed, handled, and played with wheeled objects and thus acquired some familiarity with the mechanical principle and affordances of wheel-and-axle technology, and they would have been equally familiar with the principal and affordances of an animal-drawn vehicle and transport. Fusing these two sets of experience and translating the combination into a new, operational technology would have required both the ability and openness to associate the two cognitively, and the time, freedom and curiosity to follow up with trial-and-error exploration at full scale. In contrast to Tripolye adults, some youngsters in this context are likely to have fulfilled all of these requirements at the same time.

I am sceptical. Even if subadults had the idea, they needed skills and the motivation, as well as control of significant material resources. They would have needed ownership and control of the ox, the tools and wood needed to make full-size wheels and axles, and a wagon to make or rebuild. That said, my scepticism is not evidence. The crucial point is that P1–P4 are all credible. Once we establish (if we can establish) a positive connection between community innovation rate and enhanced material scaffolds of subadult learning, we need ways of testing between these four possibilities.

Judging from her paper title, ‘Learner-driven innovation in the stone tool technology of early *Homo sapiens*’, Wilkins (2020) also supports the idea that children invent procedures that are new to their community. However, the paper is more on H3: it is on subadult generation of variation rather than of adaptive novelties. Wilkins argues that archaeologists have over-estimated both the prevalence and the importance of high-fidelity vertical social learning. Her paper argues that hominin learners were likely to use a variety of learning strategies, often combining aspects of individual and social learning, for young humans are and were willing to experiment and play. In particular, emulation was probably an important mode of social learning, and this will typically result in variation in method, even when outcomes remain fairly similar. I think Wilkins is right to draw more attention to the variety of social learning strategies and their role in generating variation. However, her analysis of variation does not distinguish between variation-as-change, and variation-as-adaptive improvement. Much variation is neutral or worse. As a consequence, her archaeological examples of variation, and of its spread in time and space, do not themselves show that ‘bottom-up’ social learning strategies find improvements and adaptive refinements. That is true even of her impressive discussion of convergence. One of her persuasive examples is of the first blade productions taking place at roughly the same time in southern Africa (Kathu Pan 1) and East Africa (Katpthurin) about 500 kyr, distant in space, and with a difference in technique. These examples of convergence do indeed show adaptive innovation, but they do not show *learner-driven* innovation. For all the record shows, innovation could be the result of adult experts refining their own technique.

Moreover, while emulation was very likely an important mode of social learning, this paper understates the difficulty of identifying social learning strategies from the lithic record. On Wilkins’ view, emulation produces functional similarity of outputs but not techniques. In contrast, high-fidelity top-down learning would produce convergence in technique as well. Wilkins identifies Middle Stone Age (MSA) sites in Ethiopia, Kenya and South Africa where there is similarity in output but not technique. She writes: ‘one would expect similarity in the reduction strategies and selective processes employed for manufacturing stone tools, but rather, intra-assemblage diversity suggests that a variety of processes were used to accomplish a single goal – a stone point suitable for use as a weapon tip. While evidence for emulation is rarely explicitly reported, several descriptions of MSA assemblages highlight core reduction strategy diversity’ (p. 5). However, this test is sound only if the stone record is the record of a single cultural community. The more it is the lumped residue of many communities, the more variation might represent between-community rather than within-community difference. Moreover, within-community convergence on technique is driven by various forms of oblique intergenerational social learning, namely conformist or prestige-based imitation. If social learning were strictly vertical, within-community differences in family technique would be preserved.

The bottom line is that, while the case for H1 is strong, and H3 plausible, the case for H4 is still to be made. Equally, it is important not to be over-sceptical about the idea that subadult experimentation is a source of adaptation, not just variation. Subadults are unlikely to refine existing tools and technology, to, say, add a shock absorber to a stone adze to reduce the chances of the head cracking in use, since a high level of expertise is needed to diagnose the problem and to identify and execute a potential solution. The same is true of recombination, as in synthesising the sled and axle technologies. However, some adaptations are finding new uses for existing technologies, and here play and experiment may well have been productive. Once cordage has been invented, it has many uses: snares, trip-wires, traps and nets as well as bindings. It is not difficult to imagine trip-wires, for example, originating in children's play.

So while it is certainly possible that subadult play and experiment find adaptive novelties, the best evidenced connection between subadult activities and innovation is captured by H2: adolescents take up and spread innovations. There is persuasive ethnographic evidence for the claim that adolescents act as innovation vectors, and good theoretical reason to expect this phenomenon to be quite general. The ethnographic evidence is reviewed in Lew-Levy et al. (2020a) and Nowell and French (2020). In one respect, the idea is uncontroversial. Out-marriage will take adolescents from their natal group into a wider world, and that will automatically result in some tendency for them to be the channel through which the distinctive ideas and practices of a residential group spread from their point of origin. Formal models underscore the importance of exporting innovation from its natal group to a broader community. Practices restricted to a residential group are very vulnerable to loss (Premo & Kuhn, 2010), and one plausible explanation of the acceleration in innovation in the late Pleistocene was the growth of social networks that enhanced this spread of innovations from their band of origin (Powell et al., 2009).

However, the ethnographic evidence, at least of the Aka and Chabu, suggests adolescents do more than aid the passive diffusion of innovations which have already become established in a residential group, however important that is. Aka and Chabu adolescents actively seek innovators as role models and prize innovation both for its social economic rewards. Ethnography supports the model of adolescents as innovation vectors. Theoretical considerations suggest that the Aka and Chabu will be typical rather than exceptional. First, there is a strong ethnographic signal that adolescent foragers have acquired a solid grounding in the life skills they need: only the most challenging skills remain to be mastered (Lew-Levy et al., 2017). So they are poised to recognise and assess innovators. Moreover, their subsistence is still subsidised by their family, so they can afford to experiment. In addition, they pay no opportunity or transition costs in opting to learn a novel rather than an established practice. They are already committed to investing time and effort into mastering one or the other, the established or the new (and perhaps they are already committed to spending social capital as well, taking on obligations of respect and deference; Henrich & Gil-White, 2001). In contrast, an adult switching practices does pay opportunity and transition costs (if, for example, a new mode of fishing requires re-equipping). So economic considerations predict that adolescents will be ready to take up innovations, whereas adults will be more conservative: they have more to pay, more to lose.

### Time to recap

On method, children's agency is not entirely veiled. It does leave traces. However, these tend to be fragile, ambiguous and fast-fading. On innovation, forager children are almost certainly more autonomous, experimental and adventurous than WEIRD children, and this was probably true of the young foragers of the early Holocene and late Pleistocene, too. Their greater willingness to experiment probably fuels a supply of variation, and perhaps occasionally adaptation as well, especially finding new uses for existing materials. Much more certainly, innovations tend to be noted, taken up and spread by adolescents.

## Data Availability

All data discussed in this paper is presented in the cited references.
